# Chloridodiphen­yl{[1-(1,3-thia­zol-2-yl-κ*N*)ethyl­idene]-4-phenyl­thio­semicarbazidato-κ^2^ *N* ^1^,*S*}tin(IV) methanol monosolvate

**DOI:** 10.1107/S1600536811037627

**Published:** 2011-09-30

**Authors:** Sri Ranjini Arumugam, Samuel S. R. Dasary, Ramaiyer Venkatraman, Hongtao Yu, Frank R. Fronczek

**Affiliations:** a Department of Chemistry and Biochemistry, Jackson State University, Jackson, MS 39217, USA; b Department of Chemistry, Louisiana State University, Baton Rouge, LA 70803, USA

## Abstract

The title compound, [Sn(C_6_H_5_)_2_(C_12_H_11_N_4_S_2_)Cl]·CH_4_O, is formed during the reaction between 2-acetyl­thia­zole 4-phenyl­thio­semicarbazone (Hacthptsc) and diphenyl­tin(IV) dichloride in methanol. In the crystal structure, the Sn atom exhibits an octa­hedral geometry with the [N_2_S] anionic tridentate thio­semicarbazone ligand having chloride *trans* to the central N and the two phenyl groups *trans* to each other. The Sn—Cl distance is 2.5929 (6), Sn—S is 2.4896 (6) and Sn—N to the central N is 2.3220 (16) Å. The MeOH mol­ecules link the Sn complexes into one-dimensional chains *via* N—H⋯O and O—H⋯Cl hydrogen bonds.

## Related literature

For the biological activity and structural characteristics of tin compounds of thio­semicarbazones, see: Teoh *et al.* (1999 ▶); Gielen *et al.* (2005 ▶); Chaudhary *et al.* (2009 ▶); Bamgboye & Bamgboye (1988 ▶); Barberi *et al.* (1993 ▶); Casas *et al.* (1994 ▶, 1996 ▶, 1997 ▶); De Sousa *et al.* (2001 ▶); Li *et al.* (2011 ▶); Macias *et al.* (1989 ▶); Huheey *et al.* (1993 ▶). For related structures, see: Venkatraman *et al.* (2004 ▶, 2007 ▶, 2009 ▶); Swesi *et al.* (2006*a* ▶,*b* ▶,*c* ▶); Sreekanth & Kurup (2004 ▶); Mendes *et al.* (2008 ▶); Li *et al.* (2011 ▶). For standard bond lengths, see: Allen *et al.* (1979 ▶); Davies (1998 ▶); Dey *et al.* (2003 ▶). For graph-set analysis, see: Etter (1990 ▶).
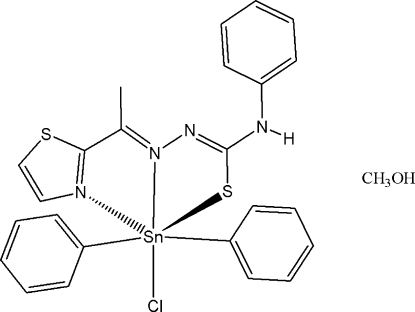


## Experimental

### 

#### Crystal data

[Sn(C_6_H_5_)_2_(C_12_H_11_N_4_S_2_)Cl]·CH_4_O
*M* *_r_* = 615.75
Monoclinic, 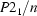

*a* = 8.5971 (10) Å
*b* = 20.182 (3) Å
*c* = 15.794 (2) Å
β = 102.050 (7)°
*V* = 2680.0 (6) Å^3^
*Z* = 4
Mo *K*α radiation
μ = 1.23 mm^−1^
*T* = 297 K
0.30 × 0.20 × 0.17 mm

#### Data collection

Nonius KappaCCD diffractometer
Absorption correction: multi-scan (*SCALEPACK*; Otwinowski & Minor, 1997 ▶) *T*_min_ = 0.709, *T*_max_ = 0.818
31922 measured reflections
8479 independent reflections
6194 reflections with *I* > 2σ(*I*)
*R*_int_ = 0.027

#### Refinement

*R*[*F* ^2^ > 2σ(*F* ^2^)] = 0.032
*wR*(*F* ^2^) = 0.070
*S* = 1.01
8479 reflections
316 parameters
H atoms treated by a mixture of independent and constrained refinement
Δρ_max_ = 0.35 e Å^−3^
Δρ_min_ = −0.58 e Å^−3^

### 

Data collection: *COLLECT* (Nonius, 2000 ▶); cell refinement: *SCALEPACK* (Otwinowski & Minor, 1997 ▶); data reduction: *DENZO* (Otwinowski & Minor, 1997 ▶) and *SCALEPACK*; program(s) used to solve structure: *SIR97* (Altomare *et al.*, 1999 ▶); program(s) used to refine structure: *SHELXL97* (Sheldrick, 2008 ▶); molecular graphics: *ORTEP-3 for Windows* (Farrugia, 1997 ▶); software used to prepare material for publication: *SHELXL97*.

## Supplementary Material

Crystal structure: contains datablock(s) global, I. DOI: 10.1107/S1600536811037627/zk2029sup1.cif

Structure factors: contains datablock(s) I. DOI: 10.1107/S1600536811037627/zk2029Isup2.hkl

Additional supplementary materials:  crystallographic information; 3D view; checkCIF report

## Figures and Tables

**Table 1:** Hydrogen-bond geometry (Å, °)

*D*—H⋯*A*	*D*—H	H⋯*A*	*D*⋯*A*	*D*—H⋯*A*
N4—H4*N*⋯O1	0.85 (2)	2.08 (2)	2.930 (3)	175 (2)
O1—H1*S*⋯Cl1^i^	0.76 (4)	2.52 (4)	3.248 (2)	162 (4)

Symmetry code: (i) 
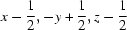
.

**Fig. 1.**
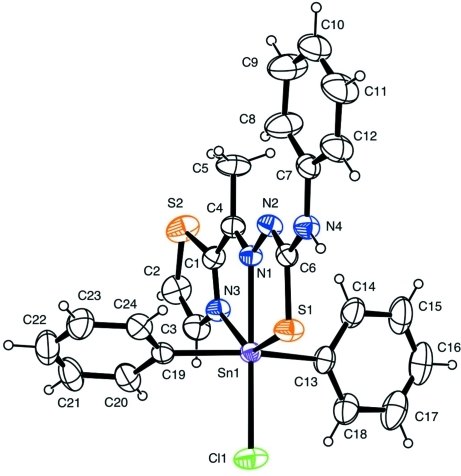
The molecular structure of the title compound. Displacement ellipsoids are drawn at the 40% probability level, and the solvent is not shown.

**Fig. 2.**
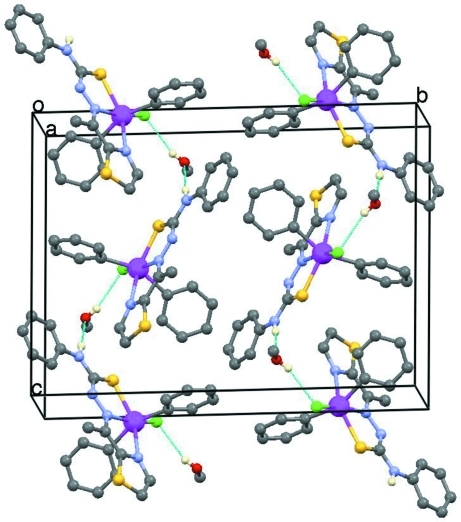
The unit cell, showing hydrogen-bonded chains.

## supplementary crystallographic information

### Comment

Metal complexes of heterocyclic thiosemicarbazones have been the subject of intensive research for the past three decades. Among the non-transitional metals, organotin(IV) based compounds received prominence due to their structural features and potent biological activity (Teoh *et al.*, 1999; Gielen *et al.*, 2005; Chaudhary *et al.*, 2009; Bamgboye & Bamgboye, 1988; Barberi *et al.*, 1993; Casas *et al.*, 1994; Casas,*et al.*, 1996; Casas *et al.*, 1997; De Sousa *et al.*, 2001; Li *et al.*, 2011). Continuing with this type of study (Venkatraman *et al.*, 2009; Venkatraman *et al.*, 2007; Swesi *et al.*, 2006*a*,*b*,*c*; Venkatraman *et al.*, 2004), we describe here the structure of a diphenyltin chloro derivative of thiazole-2-carbaldehyde *N*(4)-phenyl-3-thiosemicarbazone.

The tin atom is coordinated by the tridentate ligand through the thiazole ring nitrogen, the azomethine nitrogen and thiolate sulfur atom. The octahedral complex also contains one chloro ligand *trans* to the central N atom of the tridentate ligand and two diphenyl groups *trans* to each other, as shown in Fig. 1. The tridentate ligand is reasonably planar, its 18 nonhydrogen atoms having a mean deviation of 0.082 Å from coplanarity, and a maximum of 0.176 (3) Å for methyl group C5. The bite angles of the 5-membered chelate rings are N1—Sn1—S1, 76.39 (4)° and N1—Sn1—N3, 67.57 (6)°. The two phenyl groups form a *trans* angle C13—Sn1—C19 154.86 (8)°, and the chloro ligand forms a *trans* angle N1—Sn1—Cl1 165.94 (4)°. The Sn—Cl bond is in the range of normal covalent radii (2.37–2.60 Å, Casas *et al.*, 1997; Davies, 1998). The Sn—C (phenyl) distances are similar to those in other tin complexes reported by us earlier(Venkatraman *et al.*, 2004; 2007; 2009; Swesi *et al.*, 2006*a*,*b*,*c*). The bond length Sn—C increases with an increase in coordination number, being longer in the title compound than in four-coordinate Ph_2_SnCl_2_ [2.122 (2) Å] and is higher than expected (Dey *et al.*, 2003). The C—S bond distance of 1.755 (2) Å is slightly shorter than a C—S single bond (1.81 Å) but longer than a C—S double bond (1.62 Å) (Macias *et al.*, 1989; Huheey *et al.*, 1993). The relatively shorter bond length of Sn—N1 (imine) (2.3322 Å) compared with Sn—N3 (thiazole) is attributed stronger base nature of thiazole nitrogen (Sreekanth & Kurup, 2004; Mendes *et al.*, 2008; Li *et al.*, 2011).

Two types of intermolecular hydrogen bonds are present, each involving both the Sn complex and the methanol solvent molecule. The amino N4—H group donates to methanol O1, and the methanol O1—H donates to the chloro ligand at 1/2 + *x*, 1/2 - *y*, 1/2 + *z*. The combination of the two hydrogen bonds forms chains of alternating Sn complexes and methanol molecules in the [1 0 1] direction, having graph set C^2^_2_(8) (Etter, 1990), as shown in Fig. 2.

### Experimental

Equimolar amounts of diphenyltin dichloride and 2-acetylthiazole 4-phenylthiosemicarbazone in dry methanol were refluxed for a period of 2 h and then allowed to cool to room temperature in presence of air. Yellow crystals of the tin complex (1) appeared in about a week.

### Refinement

All H atoms on C were placed in calculated positions, guided by difference maps, with C—H bond distances 0.93–0.96 Å. N—H and solvent O—H hydrogen coordinates were refined. Displacement parameters for H atoms were assigned as *U*_iso_=1.2*U*_eq_ (1.5 for Me and OH). A torsional parameter was refined for each methyl group.

### Crystal data

**Table:**

[Sn(C_6_H_5_)_2_(C_12_H_11_N_4_S_2_)Cl]·CH_4_O	*F*(000) = 1240
*M**_r_* = 615.75	*D*_x_ = 1.526 Mg m^−^^3^
Monoclinic, *P*2_1_/*n*	Mo *K*α radiation, λ = 0.71073 Å
Hall symbol: -P 2yn	Cell parameters from 7958 reflections
*a* = 8.5971 (10) Å	θ = 2.5–32.0°
*b* = 20.182 (3) Å	µ = 1.23 mm^−^^1^
*c* = 15.794 (2) Å	*T* = 297 K
β = 102.050 (7)°	Fragment, yellow
*V* = 2680.0 (6) Å^3^	0.30 × 0.20 × 0.17 mm
*Z* = 4	

### Data collection

**Table:**

Nonius KappaCCD diffractometer	8479 independent reflections
Radiation source: fine-focus sealed tube	6194 reflections with *I* > 2σ(*I*)
graphite	*R*_int_ = 0.027
ω and φ scans	θ_max_ = 32.0°, θ_min_ = 2.6°
Absorption correction: multi-scan (*SCALEPACK*; Otwinowski & Minor, 1997)	*h* = −12→12
*T*_min_ = 0.709, *T*_max_ = 0.818	*k* = −28→25
31922 measured reflections	*l* = −23→23

### Refinement

**Table:**

Refinement on *F*^2^	Secondary atom site location: difference Fourier map
Least-squares matrix: full	Hydrogen site location: inferred from neighbouring sites
*R*[*F*^2^ > 2σ(*F*^2^)] = 0.032	H atoms treated by a mixture of independent and constrained refinement
*wR*(*F*^2^) = 0.070	*w* = 1/[σ^2^(*F*_o_^2^) + (0.0236*P*)^2^ + 0.9273*P*] where *P* = (*F*_o_^2^ + 2*F*_c_^2^)/3
*S* = 1.01	(Δ/σ)_max_ = 0.002
8479 reflections	Δρ_max_ = 0.35 e Å^−^^3^
316 parameters	Δρ_min_ = −0.58 e Å^−^^3^
0 restraints	Extinction correction: *SHELXL97* (Sheldrick, 2008), Fc^*^=kFc[1+0.001xFc^2^λ^3^/sin(2θ)]^-1/4^
Primary atom site location: structure-invariant direct methods	Extinction coefficient: 0.00176 (19)

### Special details

**Table:**

Geometry. All e.s.d.'s (except the e.s.d. in the dihedral angle between two l.s. planes) are estimated using the full covariance matrix. The cell e.s.d.'s are taken into account individually in the estimation of e.s.d.'s in distances, angles and torsion angles; correlations between e.s.d.'s in cell parameters are only used when they are defined by crystal symmetry. An approximate (isotropic) treatment of cell e.s.d.'s is used for estimating e.s.d.'s involving l.s. planes.
Refinement. Refinement of *F*^2^ against ALL reflections. The weighted *R*-factor *wR* and goodness of fit *S* are based on *F*^2^, conventional *R*-factors *R* are based on *F*, with *F* set to zero for negative *F*^2^. The threshold expression of *F*^2^ > σ(*F*^2^) is used only for calculating *R*-factors(gt) *etc*. and is not relevant to the choice of reflections for refinement. *R*-factors based on *F*^2^ are statistically about twice as large as those based on *F*, and *R*- factors based on ALL data will be even larger.

### Fractional atomic coordinates and isotropic or equivalent isotropic displacement parameters (Å^2^)

**Table:**

	*x*	*y*	*z*	*U*_iso_*/*U*_eq_	
Sn1	0.400041 (15)	0.263309 (7)	0.526902 (8)	0.03515 (5)	
Cl1	0.66803 (7)	0.21480 (4)	0.50486 (4)	0.06294 (17)	
S1	0.35758 (7)	0.31789 (3)	0.38254 (3)	0.04796 (14)	
S2	0.09795 (9)	0.29250 (4)	0.75637 (4)	0.06512 (19)	
N1	0.17015 (18)	0.32507 (8)	0.52098 (10)	0.0356 (4)	
N2	0.10480 (19)	0.36198 (9)	0.44956 (11)	0.0403 (4)	
N3	0.2829 (2)	0.24841 (9)	0.66321 (12)	0.0419 (4)	
N4	0.1258 (2)	0.39796 (10)	0.31437 (12)	0.0426 (4)	
H4N	0.175 (3)	0.3937 (12)	0.2732 (15)	0.051*	
C1	0.1632 (2)	0.28859 (11)	0.66081 (13)	0.0396 (4)	
C2	0.2388 (3)	0.23643 (14)	0.80026 (17)	0.0615 (7)	
H2	0.2540	0.2202	0.8565	0.074*	
C3	0.3249 (3)	0.21897 (13)	0.74245 (15)	0.0540 (6)	
H3	0.4080	0.1887	0.7554	0.065*	
C4	0.0950 (2)	0.32769 (11)	0.58439 (13)	0.0402 (5)	
C5	−0.0537 (3)	0.36652 (15)	0.58116 (17)	0.0617 (7)	
H5A	−0.0412	0.4101	0.5590	0.092*	
H5B	−0.0746	0.3700	0.6384	0.092*	
H5C	−0.1410	0.3445	0.5440	0.092*	
C6	0.1826 (2)	0.36139 (10)	0.38687 (13)	0.0367 (4)	
C7	−0.0056 (2)	0.44193 (11)	0.29689 (14)	0.0430 (5)	
C8	−0.0943 (4)	0.46150 (16)	0.35598 (19)	0.0756 (9)	
H8	−0.0706	0.4451	0.4122	0.091*	
C9	−0.2185 (4)	0.50555 (18)	0.3312 (2)	0.0845 (10)	
H9	−0.2779	0.5181	0.3715	0.101*	
C10	−0.2564 (3)	0.53100 (16)	0.2506 (2)	0.0739 (8)	
H10	−0.3395	0.5611	0.2354	0.089*	
C11	−0.1695 (3)	0.51142 (17)	0.1920 (2)	0.0796 (9)	
H11	−0.1941	0.5281	0.1360	0.096*	
C12	−0.0455 (3)	0.46717 (15)	0.21483 (17)	0.0631 (7)	
H12	0.0118	0.4543	0.1738	0.076*	
C13	0.2818 (3)	0.17040 (12)	0.49558 (13)	0.0445 (5)	
C14	0.1166 (3)	0.16993 (15)	0.47549 (18)	0.0676 (8)	
H14	0.0612	0.2091	0.4787	0.081*	
C15	0.0335 (4)	0.11204 (19)	0.4508 (2)	0.0847 (10)	
H15	−0.0771	0.1127	0.4377	0.102*	
C16	0.1118 (5)	0.05441 (17)	0.4454 (2)	0.0839 (10)	
H16	0.0553	0.0156	0.4288	0.101*	
C17	0.2748 (5)	0.05366 (15)	0.4646 (2)	0.0842 (10)	
H17	0.3289	0.0142	0.4609	0.101*	
C18	0.3598 (4)	0.11156 (13)	0.48954 (17)	0.0652 (7)	
H18	0.4704	0.1105	0.5023	0.078*	
C19	0.5337 (2)	0.33492 (11)	0.61156 (13)	0.0408 (5)	
C20	0.6194 (3)	0.31871 (15)	0.69410 (15)	0.0557 (6)	
H20	0.6246	0.2749	0.7127	0.067*	
C21	0.6963 (3)	0.36751 (19)	0.74819 (17)	0.0714 (9)	
H21	0.7518	0.3564	0.8034	0.086*	
C22	0.6916 (3)	0.43192 (19)	0.7215 (2)	0.0784 (10)	
H22	0.7428	0.4645	0.7588	0.094*	
C23	0.6124 (3)	0.44858 (15)	0.6407 (2)	0.0732 (8)	
H23	0.6114	0.4924	0.6223	0.088*	
C24	0.5321 (3)	0.39994 (13)	0.58489 (17)	0.0544 (6)	
H24	0.4776	0.4116	0.5297	0.065*	
O1	0.2782 (3)	0.38699 (13)	0.16545 (14)	0.0795 (7)	
H1S	0.252 (5)	0.357 (2)	0.137 (3)	0.119*	
C25	0.4439 (4)	0.38909 (18)	0.1816 (2)	0.0845 (10)	
H25A	0.4819	0.4219	0.2250	0.127*	
H25B	0.4780	0.4003	0.1292	0.127*	
H25C	0.4860	0.3465	0.2016	0.127*	

### Atomic displacement parameters (Å^2^)

**Table:**

	*U*^11^	*U*^22^	*U*^33^	*U*^12^	*U*^13^	*U*^23^
Sn1	0.03442 (8)	0.03828 (8)	0.03287 (8)	0.00389 (6)	0.00732 (5)	−0.00039 (6)
Cl1	0.0524 (3)	0.0723 (4)	0.0704 (4)	0.0224 (3)	0.0273 (3)	0.0048 (3)
S1	0.0483 (3)	0.0618 (4)	0.0370 (3)	0.0164 (3)	0.0163 (2)	0.0096 (2)
S2	0.0809 (4)	0.0739 (5)	0.0516 (4)	0.0144 (4)	0.0391 (3)	0.0095 (3)
N1	0.0337 (8)	0.0375 (9)	0.0366 (9)	0.0025 (7)	0.0096 (7)	0.0025 (7)
N2	0.0351 (8)	0.0463 (11)	0.0394 (9)	0.0050 (7)	0.0072 (7)	0.0068 (8)
N3	0.0436 (9)	0.0452 (11)	0.0387 (10)	0.0031 (8)	0.0129 (8)	0.0029 (7)
N4	0.0435 (10)	0.0478 (11)	0.0363 (10)	0.0051 (8)	0.0078 (8)	0.0054 (8)
C1	0.0424 (10)	0.0421 (11)	0.0380 (11)	−0.0025 (9)	0.0171 (9)	−0.0008 (9)
C2	0.0757 (17)	0.0685 (18)	0.0436 (13)	0.0027 (14)	0.0202 (12)	0.0133 (12)
C3	0.0571 (14)	0.0582 (16)	0.0458 (13)	0.0055 (12)	0.0089 (11)	0.0108 (11)
C4	0.0361 (10)	0.0438 (12)	0.0431 (11)	−0.0001 (9)	0.0137 (9)	0.0022 (9)
C5	0.0471 (13)	0.0786 (19)	0.0665 (17)	0.0212 (13)	0.0285 (12)	0.0139 (14)
C6	0.0356 (10)	0.0370 (11)	0.0359 (10)	−0.0009 (8)	0.0038 (8)	0.0009 (8)
C7	0.0411 (11)	0.0379 (12)	0.0463 (12)	−0.0010 (9)	0.0004 (9)	0.0034 (10)
C8	0.086 (2)	0.079 (2)	0.0632 (18)	0.0403 (17)	0.0187 (15)	0.0179 (15)
C9	0.084 (2)	0.088 (2)	0.083 (2)	0.0415 (18)	0.0211 (17)	0.0145 (19)
C10	0.0612 (16)	0.0656 (19)	0.085 (2)	0.0173 (14)	−0.0066 (15)	0.0078 (16)
C11	0.0730 (19)	0.090 (2)	0.0663 (19)	0.0237 (17)	−0.0076 (15)	0.0245 (17)
C12	0.0585 (14)	0.0755 (19)	0.0516 (15)	0.0139 (13)	0.0030 (12)	0.0135 (13)
C13	0.0581 (13)	0.0454 (13)	0.0306 (10)	−0.0060 (10)	0.0104 (9)	−0.0026 (9)
C14	0.0623 (16)	0.0642 (18)	0.080 (2)	−0.0180 (13)	0.0245 (14)	−0.0305 (15)
C15	0.083 (2)	0.093 (3)	0.084 (2)	−0.041 (2)	0.0294 (17)	−0.0394 (19)
C16	0.129 (3)	0.066 (2)	0.0580 (18)	−0.045 (2)	0.0219 (19)	−0.0142 (15)
C17	0.143 (3)	0.0404 (16)	0.0615 (19)	−0.0015 (18)	0.0033 (19)	−0.0003 (13)
C18	0.0880 (19)	0.0441 (15)	0.0547 (15)	0.0070 (14)	−0.0050 (13)	0.0002 (12)
C19	0.0299 (9)	0.0526 (14)	0.0417 (11)	−0.0009 (9)	0.0115 (8)	−0.0092 (10)
C20	0.0470 (12)	0.0766 (18)	0.0439 (13)	−0.0096 (12)	0.0106 (10)	−0.0023 (12)
C21	0.0528 (15)	0.119 (3)	0.0429 (14)	−0.0235 (16)	0.0124 (11)	−0.0221 (16)
C22	0.0642 (17)	0.098 (3)	0.077 (2)	−0.0290 (17)	0.0238 (16)	−0.0406 (19)
C23	0.0661 (17)	0.0568 (18)	0.098 (2)	−0.0130 (14)	0.0212 (16)	−0.0207 (16)
C24	0.0479 (12)	0.0528 (15)	0.0614 (15)	0.0000 (11)	0.0089 (11)	−0.0047 (12)
O1	0.0758 (14)	0.1003 (19)	0.0638 (14)	0.0098 (13)	0.0178 (11)	−0.0101 (11)
C25	0.084 (2)	0.095 (3)	0.080 (2)	−0.0011 (19)	0.0276 (17)	0.0158 (18)

### Geometric parameters (Å, °)

**Table:**

Sn1—C19	2.134 (2)	C10—H10	0.9300
Sn1—C13	2.141 (2)	C11—C12	1.379 (4)
Sn1—N1	2.3220 (16)	C11—H11	0.9300
Sn1—S1	2.4896 (6)	C12—H12	0.9300
Sn1—N3	2.5779 (18)	C13—C18	1.377 (3)
Sn1—Cl1	2.5929 (6)	C13—C14	1.389 (3)
S1—C6	1.755 (2)	C14—C15	1.383 (4)
S2—C2	1.696 (3)	C14—H14	0.9300
S2—C1	1.718 (2)	C15—C16	1.355 (5)
N1—C4	1.301 (2)	C15—H15	0.9300
N1—N2	1.371 (2)	C16—C17	1.371 (5)
N2—C6	1.306 (2)	C16—H16	0.9300
N3—C1	1.304 (3)	C17—C18	1.391 (4)
N3—C3	1.364 (3)	C17—H17	0.9300
N4—C6	1.364 (3)	C18—H18	0.9300
N4—C7	1.418 (3)	C19—C24	1.377 (3)
N4—H4N	0.85 (2)	C19—C20	1.396 (3)
C1—C4	1.459 (3)	C20—C21	1.379 (4)
C2—C3	1.337 (3)	C20—H20	0.9300
C2—H2	0.9300	C21—C22	1.364 (5)
C3—H3	0.9300	C21—H21	0.9300
C4—C5	1.491 (3)	C22—C23	1.358 (4)
C5—H5A	0.9600	C22—H22	0.9300
C5—H5B	0.9600	C23—C24	1.401 (4)
C5—H5C	0.9600	C23—H23	0.9300
C7—C12	1.368 (3)	C24—H24	0.9300
C7—C8	1.380 (3)	O1—C25	1.395 (4)
C8—C9	1.382 (4)	O1—H1S	0.76 (4)
C8—H8	0.9300	C25—H25A	0.9600
C9—C10	1.348 (4)	C25—H25B	0.9600
C9—H9	0.9300	C25—H25C	0.9600
C10—C11	1.363 (4)		
			
C19—Sn1—C13	154.86 (8)	C10—C9—H9	118.9
C19—Sn1—N1	90.24 (7)	C8—C9—H9	118.9
C13—Sn1—N1	95.85 (8)	C9—C10—C11	118.1 (3)
C19—Sn1—S1	103.35 (6)	C9—C10—H10	120.9
C13—Sn1—S1	101.78 (6)	C11—C10—H10	120.9
N1—Sn1—S1	76.39 (4)	C10—C11—C12	120.9 (3)
C19—Sn1—N3	78.98 (7)	C10—C11—H11	119.5
C13—Sn1—N3	80.88 (7)	C12—C11—H11	119.5
N1—Sn1—N3	67.57 (6)	C7—C12—C11	121.0 (3)
S1—Sn1—N3	143.93 (4)	C7—C12—H12	119.5
C19—Sn1—Cl1	87.85 (5)	C11—C12—H12	119.5
C13—Sn1—Cl1	91.74 (6)	C18—C13—C14	117.9 (2)
N1—Sn1—Cl1	165.94 (4)	C18—C13—Sn1	123.85 (18)
S1—Sn1—Cl1	90.49 (2)	C14—C13—Sn1	118.16 (19)
N3—Sn1—Cl1	125.54 (4)	C15—C14—C13	120.9 (3)
C6—S1—Sn1	98.55 (7)	C15—C14—H14	119.5
C2—S2—C1	89.55 (12)	C13—C14—H14	119.5
C4—N1—N2	115.29 (16)	C16—C15—C14	120.6 (3)
C4—N1—Sn1	123.10 (14)	C16—C15—H15	119.7
N2—N1—Sn1	121.61 (11)	C14—C15—H15	119.7
C6—N2—N1	115.61 (16)	C15—C16—C17	119.6 (3)
C1—N3—C3	110.72 (19)	C15—C16—H16	120.2
C1—N3—Sn1	110.27 (13)	C17—C16—H16	120.2
C3—N3—Sn1	137.91 (15)	C16—C17—C18	120.4 (3)
C6—N4—C7	129.29 (18)	C16—C17—H17	119.8
C6—N4—H4N	115.9 (16)	C18—C17—H17	119.8
C7—N4—H4N	114.8 (16)	C13—C18—C17	120.6 (3)
N3—C1—C4	122.62 (17)	C13—C18—H18	119.7
N3—C1—S2	113.78 (16)	C17—C18—H18	119.7
C4—C1—S2	123.59 (16)	C24—C19—C20	118.6 (2)
C3—C2—S2	110.3 (2)	C24—C19—Sn1	118.94 (17)
C3—C2—H2	124.9	C20—C19—Sn1	122.45 (19)
S2—C2—H2	124.9	C21—C20—C19	120.2 (3)
C2—C3—N3	115.7 (2)	C21—C20—H20	119.9
C2—C3—H3	122.2	C19—C20—H20	119.9
N3—C3—H3	122.2	C22—C21—C20	120.7 (3)
N1—C4—C1	115.86 (18)	C22—C21—H21	119.7
N1—C4—C5	123.70 (19)	C20—C21—H21	119.7
C1—C4—C5	120.43 (18)	C23—C22—C21	120.2 (3)
C4—C5—H5A	109.5	C23—C22—H22	119.9
C4—C5—H5B	109.5	C21—C22—H22	119.9
H5A—C5—H5B	109.5	C22—C23—C24	120.2 (3)
C4—C5—H5C	109.5	C22—C23—H23	119.9
H5A—C5—H5C	109.5	C24—C23—H23	119.9
H5B—C5—H5C	109.5	C19—C24—C23	120.2 (3)
N2—C6—N4	118.77 (18)	C19—C24—H24	119.9
N2—C6—S1	127.81 (16)	C23—C24—H24	119.9
N4—C6—S1	113.42 (15)	C25—O1—H1S	107 (3)
C12—C7—C8	118.0 (2)	O1—C25—H25A	109.5
C12—C7—N4	116.7 (2)	O1—C25—H25B	109.5
C8—C7—N4	125.3 (2)	H25A—C25—H25B	109.5
C7—C8—C9	119.7 (3)	O1—C25—H25C	109.5
C7—C8—H8	120.1	H25A—C25—H25C	109.5
C9—C8—H8	120.1	H25B—C25—H25C	109.5
C10—C9—C8	122.2 (3)		
			
C19—Sn1—S1—C6	−87.39 (9)	Sn1—S1—C6—N2	−0.3 (2)
C13—Sn1—S1—C6	92.81 (9)	Sn1—S1—C6—N4	179.95 (14)
N1—Sn1—S1—C6	−0.44 (8)	C6—N4—C7—C12	−173.6 (2)
N3—Sn1—S1—C6	2.17 (11)	C6—N4—C7—C8	7.1 (4)
Cl1—Sn1—S1—C6	−175.30 (7)	C12—C7—C8—C9	−0.3 (5)
C19—Sn1—N1—C4	−74.56 (18)	N4—C7—C8—C9	179.0 (3)
C13—Sn1—N1—C4	81.01 (17)	C7—C8—C9—C10	−0.5 (6)
S1—Sn1—N1—C4	−178.25 (17)	C8—C9—C10—C11	0.9 (6)
N3—Sn1—N1—C4	3.41 (16)	C9—C10—C11—C12	−0.4 (5)
Cl1—Sn1—N1—C4	−156.65 (15)	C8—C7—C12—C11	0.7 (4)
C19—Sn1—N1—N2	104.97 (15)	N4—C7—C12—C11	−178.6 (3)
C13—Sn1—N1—N2	−99.46 (15)	C10—C11—C12—C7	−0.4 (5)
S1—Sn1—N1—N2	1.28 (14)	C19—Sn1—C13—C18	−76.4 (3)
N3—Sn1—N1—N2	−177.06 (16)	N1—Sn1—C13—C18	−179.58 (19)
Cl1—Sn1—N1—N2	22.9 (3)	S1—Sn1—C13—C18	103.14 (19)
C4—N1—N2—C6	177.74 (19)	N3—Sn1—C13—C18	−113.5 (2)
Sn1—N1—N2—C6	−1.8 (2)	Cl1—Sn1—C13—C18	12.27 (19)
C19—Sn1—N3—C1	88.85 (16)	C19—Sn1—C13—C14	107.3 (2)
C13—Sn1—N3—C1	−106.27 (16)	N1—Sn1—C13—C14	4.12 (19)
N1—Sn1—N3—C1	−6.02 (14)	S1—Sn1—C13—C14	−73.16 (18)
S1—Sn1—N3—C1	−8.76 (19)	N3—Sn1—C13—C14	70.24 (18)
Cl1—Sn1—N3—C1	168.14 (13)	Cl1—Sn1—C13—C14	−164.03 (18)
C19—Sn1—N3—C3	−77.5 (2)	C18—C13—C14—C15	0.4 (4)
C13—Sn1—N3—C3	87.4 (2)	Sn1—C13—C14—C15	176.9 (2)
N1—Sn1—N3—C3	−172.3 (2)	C13—C14—C15—C16	−0.2 (5)
S1—Sn1—N3—C3	−175.08 (19)	C14—C15—C16—C17	−0.1 (5)
Cl1—Sn1—N3—C3	1.8 (2)	C15—C16—C17—C18	0.1 (5)
C3—N3—C1—C4	178.9 (2)	C14—C13—C18—C17	−0.4 (4)
Sn1—N3—C1—C4	8.7 (3)	Sn1—C13—C18—C17	−176.7 (2)
C3—N3—C1—S2	0.5 (2)	C16—C17—C18—C13	0.2 (5)
Sn1—N3—C1—S2	−169.73 (10)	C13—Sn1—C19—C24	−161.17 (19)
C2—S2—C1—N3	−0.6 (2)	N1—Sn1—C19—C24	−56.77 (17)
C2—S2—C1—C4	−179.0 (2)	S1—Sn1—C19—C24	19.30 (17)
C1—S2—C2—C3	0.5 (2)	N3—Sn1—C19—C24	−123.84 (17)
S2—C2—C3—N3	−0.3 (3)	Cl1—Sn1—C19—C24	109.30 (16)
C1—N3—C3—C2	−0.1 (3)	C13—Sn1—C19—C20	17.0 (3)
Sn1—N3—C3—C2	166.14 (19)	N1—Sn1—C19—C20	121.42 (17)
N2—N1—C4—C1	179.97 (17)	S1—Sn1—C19—C20	−162.51 (16)
Sn1—N1—C4—C1	−0.5 (3)	N3—Sn1—C19—C20	54.35 (16)
N2—N1—C4—C5	1.2 (3)	Cl1—Sn1—C19—C20	−72.51 (16)
Sn1—N1—C4—C5	−179.29 (18)	C24—C19—C20—C21	2.0 (3)
N3—C1—C4—N1	−6.4 (3)	Sn1—C19—C20—C21	−176.23 (17)
S2—C1—C4—N1	171.93 (17)	C19—C20—C21—C22	−0.9 (4)
N3—C1—C4—C5	172.5 (2)	C20—C21—C22—C23	−0.8 (4)
S2—C1—C4—C5	−9.2 (3)	C21—C22—C23—C24	1.4 (4)
N1—N2—C6—N4	−178.91 (18)	C20—C19—C24—C23	−1.4 (3)
N1—N2—C6—S1	1.4 (3)	Sn1—C19—C24—C23	176.88 (18)
C7—N4—C6—N2	4.4 (3)	C22—C23—C24—C19	−0.3 (4)
C7—N4—C6—S1	−175.87 (18)		

### Hydrogen-bond geometry (Å, °)

**Table:**

*D*—H···*A*	*D*—H	H···*A*	*D*···*A*	*D*—H···*A*
N4—H4N···O1	0.85 (2)	2.08 (2)	2.930 (3)	175 (2)
O1—H1S···Cl1^i^	0.76 (4)	2.52 (4)	3.248 (2)	162 (4)

Symmetry codes: (i) *x*−1/2, −*y*+1/2, *z*−1/2.

## References

[bb1] Allen, F. H., Bellard, S., Brice, M. D., Cartwright, B. A., Doubleday, A., Higgs, H., Hummelink, T., Hummelink-Peters, B. G., Kennard, O., Motherwell, W. D. S., Rodgers, J. R. & Watson, D. G. (1979). *Acta Cryst.* B**35**, 2331–2339.

[bb2] Altomare, A., Burla, M. C., Camalli, M., Cascarano, G. L., Giacovazzo, C., Guagliardi, A., Moliterni, A. G. G., Polidori, G. & Spagna, R. (1999). *J. Appl. Cryst.* **32**, 115–119.

[bb3] Bamgboye, T. T. & Bamgboye, O. A. (1988). *Inorg. Chim. Acta*, **144**, 249–252.

[bb4] Barberi, R. S., Beraldo, H. O., Filgueiras, C. A. L., Abras, A., Nixon, J. F. & Hitchcock, P. B. (1993). *Inorg. Chim. Acta*, **206**, 169–172.

[bb5] Casas, J. S., Castineiras, A., Couce, M. D., Martinez, G., Sordo, J. & Varela, J. M. (1996). *J. Organomet. Chem.* **517**, 165–172.

[bb6] Casas, J. S., Castineiras, A., Martinez, E. G., Gonzalez, A. S., Sanchez, A. & Sordo, J. (1997). *Polyhedron*, **16**, 795–800.

[bb7] Casas, J. S., Castineiras, A., Sanchez, A., Sordo, J., Vazquez-Lopez, A., Rodriguez-Argiuelles, M. C. & Russo. U. (1994). *Inorg. Chim. Acta*, **221**, 61–68.

[bb8] Chaudhary, P., Swami, M., Sharma, D. K. & Singh, R. V. (2009). *Appl. Organomet. Chem.* **23**, 140–149.

[bb9] Davies, A. G. (1998). *Radical Chemistry of Tin*, 2nd ed., edited by P. J. Smith, pp. 265–289. London: Blackie.

[bb10] De Sousa, G. F., Francisco, R. H. P., Gambardella, M. T. P., Santos, R. H. A. & Abras, A. (2001). *J. Braz. Chem. Soc.* **12**, 722–728.

[bb11] Dey, D. K., Samanta, B., Lycka, A. & Dahlenburg, L. (2003). *Z. Naturforsch. Teil B*, **58**, 336–344.

[bb12] Etter, M. C. (1990). *Acc. Chem. Res.* **23**, 120–126.

[bb13] Farrugia, L. J. (1997). *J. Appl. Cryst.* **30**, 565.

[bb14] Gielen, M., Biesemans, R. & Willen, R. (2005). *Appl. Organomet. Chem.* **19**, 440–450.

[bb15] Huheey, J. E., Keiter, E. A. & Keitar, R. L. (1993). *Inorganic Chemistry. Principles of Structure and Reactivity*, 4th ed. New York: Harper Collins.

[bb16] Li, M. X., Zhang, D., Zhang, L. Z., Niu, J. Y. & Ji, B. S. (2011). *J. Organomet. Chem.* **696**, 852–858.

[bb17] Macias, A., Rodriguez-Arguelles, M. C., Suarez, M. I., Casas, J. S. & Sordo, J. (1989). *J. Chem. Soc. Dalton Trans.* pp. 1787–1791.

[bb18] Mendes, I. C., Moreira, J. P., Ardission, J. D., dos Santos, R. G., da Silva, P. R. O., Garcia, I., Castineiras, A. & Beraldo, H. (2008). *J. Med. Chem.* **43**, 1454–1461. 10.1016/j.ejmech.2007.09.016 17983689

[bb19] Nonius (2000). *COLLECT.* Nonius BV, Delft, The Netherlands.

[bb20] Otwinowski, Z. & Minor, W. (1997). *Methods in Enzymology*, Vol. 276, *Macromolecular Crystallography*, Part A, edited by C. W. Carter Jr & R. M. Sweet, pp. 307–326. New York: Academic Press.

[bb21] Sheldrick, G. M. (2008). *Acta Cryst.* A**64**, 112–122. 10.1107/S0108767307043930 18156677

[bb22] Sreekanth, A. & Kurup, M. R. P. (2004). *Polyhedron*, **23**, 969–978.

[bb23] Swesi, A. T., Farina, Y., Venkatraman, R. & Ng, S. W. (2006*a*). *Acta Cryst.* E**62**, m3016–m3017.

[bb24] Swesi, A. T., Farina, Y., Venkatraman, R. & Ng, S. W. (2006*b*). *Acta Cryst.* E**62**, m3018–m3019.

[bb25] Swesi, A. T., Farina, Y., Venkatraman, R. & Ng, S. W. (2006*c*). *Acta Cryst.* E**62**, m3020–m3021.

[bb26] Teoh, S. G., Ang, S. H. & Ong, C. W. (1999). *J. Organomet. Chem.* **580**, 17–21.

[bb27] Venkatraman, R., Ray, P. C. & Fronczek, F. R. (2004). *Acta Cryst.* E**60**, m1035–m1037. 10.1107/S0108270104015963 15345840

[bb28] Venkatraman, R., Sitole, L., Adams, T. D., Cameron, J. A. & Fronczek, F. R. (2007). *Acta Cryst.* E**63**, m2212–m2213.

[bb29] Venkatraman, R., Sitole, L. & Fronczek, F. R. (2009). *Acta Cryst.* E**65**, m1653–m1654. 10.1107/S1600536809047400 PMC2971835 21578665

